# Author Correction: Broadly cross-reactive human antibodies that inhibit genogroup I and II noroviruses

**DOI:** 10.1038/s41467-021-26418-1

**Published:** 2021-10-14

**Authors:** Gabriela Alvarado, Wilhelm Salmen, Khalil Ettayebi, Liya Hu, Banumathi Sankaran, Mary K. Estes, B. V. Venkataram Prasad, James E. Crowe

**Affiliations:** 1 Department of Pathology, Microbiology and Immunology, Vanderbilt University Medical Center, Nashville, TN USA; 2 Department of Molecular Virology and Microbiology, Baylor College of Medicine, Houston, TX USA; 3 The Verna and Marrs McLean Department of Biochemistry and Molecular Biology, Baylor College of Medicine, Houston, TX USA; 4 Berkeley Center for Structural Biology, Molecular Biophysics and Integrated Bioimaging, Lawrence Berkeley Laboratory, Berkeley, CA USA; 5 Department of Medicine-Gastroenterology and Hepatology, Baylor College of Medicine, Houston, TX USA; 6 The Vanderbilt Vaccine Center, Vanderbilt University Medical Center, Nashville, TN USA; 7 Department of Pediatrics, Vanderbilt University Medical Center, Nashville, TN USA

**Keywords:** X-ray crystallography, Antibodies, Viral host response

Correction to: *Nature Communications* 10.1038/s41467-021-24649-w, published online 14 July 2021.

The original version of this Article contained an error in Fig. 5B, in which the header was incorrectly given as ‘GII.17 _ Fab’, rather than the correct ‘GII.4 _ Fab’. This has been corrected in both the PDF and HTML versions of the Article.

