# Supplementary material for: Broadly cross-reactive human antibodies that inhibit genogroup I and II noroviruses
Source: Nat Commun. 2021 Jul 14;12:4320. doi: 10.1038/s41467-021-24649-w (PMC8280134; doi:10.1038/s41467-021-24649-w)
Supplement: Supplementary file 1 — Supplementary Information [file 41467_2021_24649_MOESM1_ESM.pdf]

SUPPLEMENTAL INFORMATION

Supplemental Figures

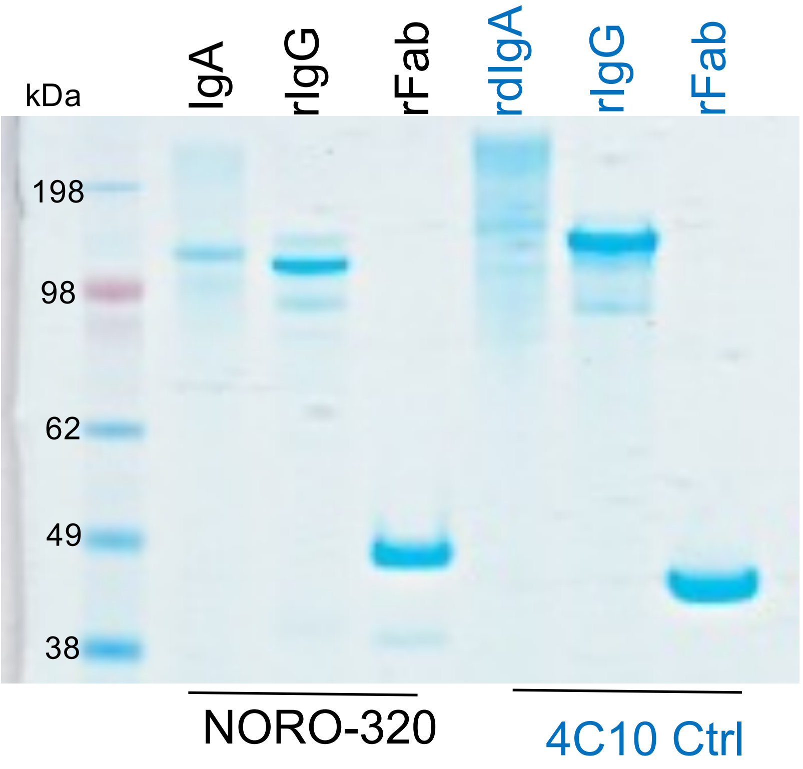

Supplemental Figure 1. Verification of molecular assembly of recombinantly expressed NORO-320 variants. NORO-320 was expressed recombinantly and purified in Fab or IgG forms. Fab, IgG and hybridoma dimeric IgA variants were resolved on an SDS-PAGE gel under non-reducing conditions. Recombinant dimeric IgA, IgG and Fab 4C10 was used as a control. This experiment was repeated independently twice.

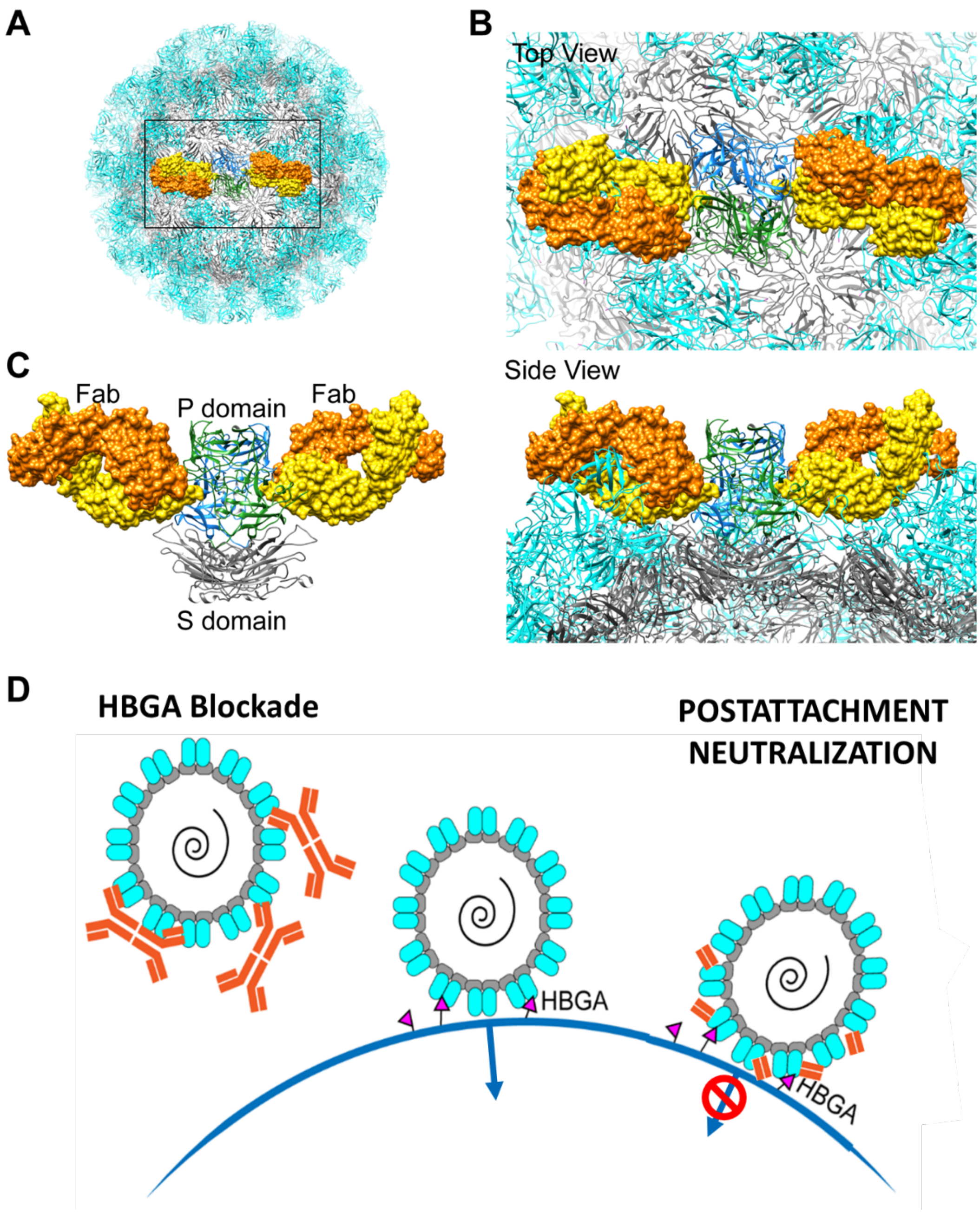

Supplemental Figure 2. Modeling of NORO-320 Fab bound to GII.4 particle. A) Superimposition of GII.4 P-domain/ NORO-320 Fab complex onto Norwalk virus capsid (PDB ID: 1IHM). P or S-domains are colored in cyan or gray, respectively. NORO-320 Fab is shown in surface representation with light or heavy chains in orange or yellow, respectively. **B-C)** Close-up views of a VP1 dimer (green and blue chains) with two molecules of NORO-320 Fab. **D)** A schematic of proposed model for the neutralization of GII strains by NORO-320 IgA or Fab.

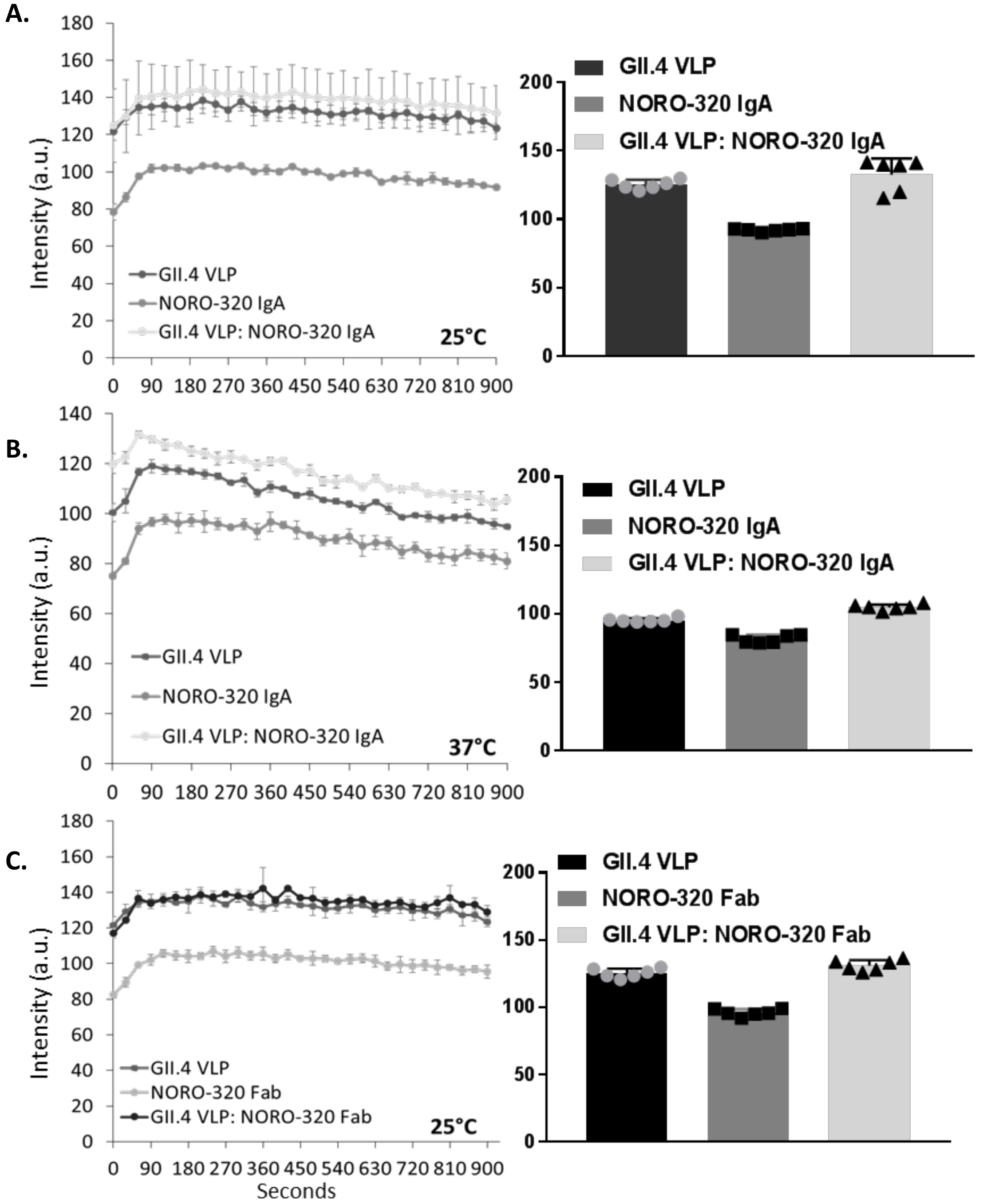

Supplemental Figure 3 *(continued on next page)*

D.

E.

F.

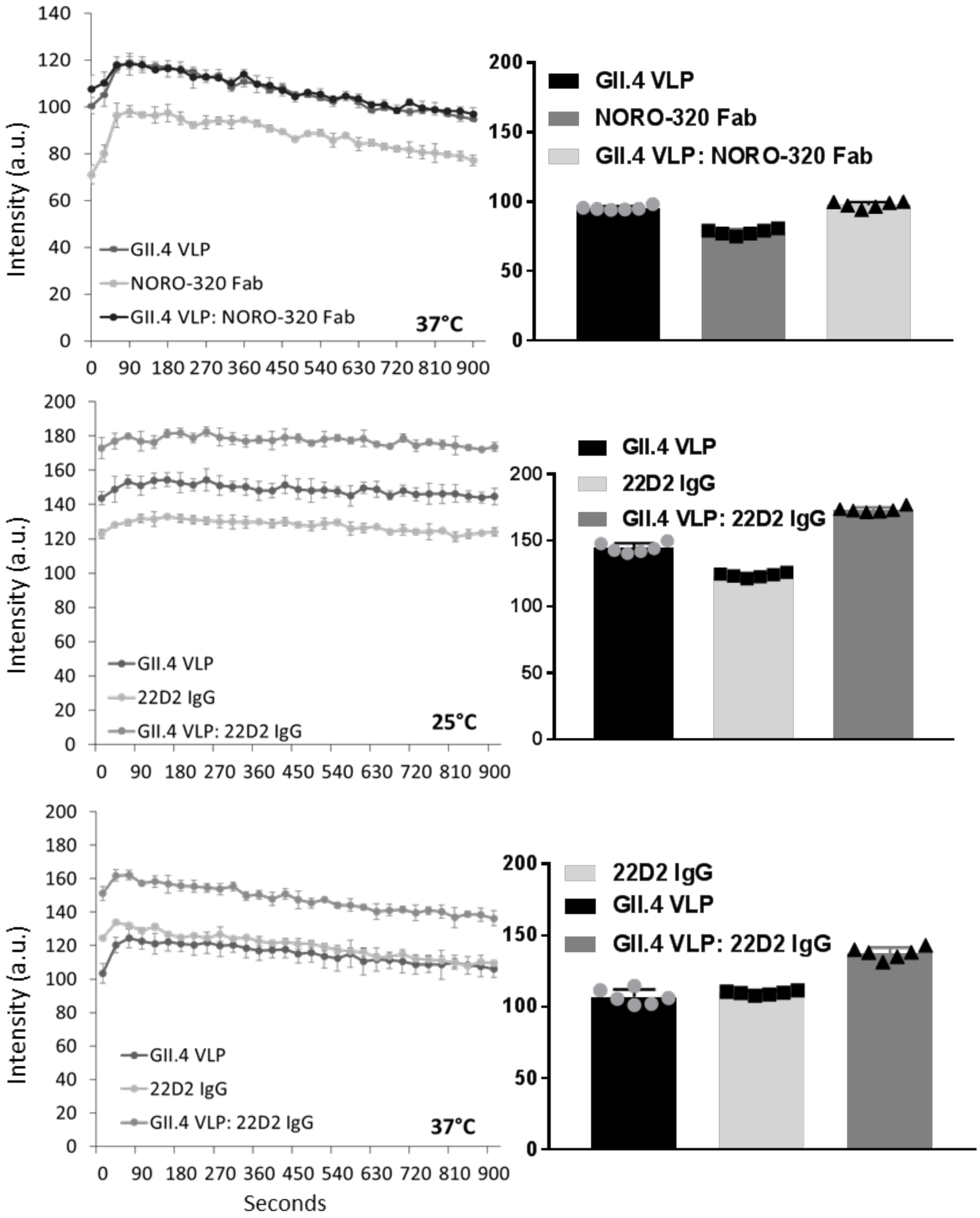

Supplemental Figure 3. Changes in bis-ANS binding upon mixing GII.4 VLP with antibody. Purified VLP (30 μg/mL, 0.5 μM concentration of the VP1) or 0.5 μM purified antibody (NORO-320 Fab, IgA, or 22D2 control) diluted in PBS buffer pH 6.0 was incubated at 25°C (A, C, E) or 37°C (B, D, F) for 10 min to allow for temperature equilibration. At time zero, bis-ANS was added to the indicated VLP, antibody, or VLP preincubated with antibody. Fluorescence was recorded every 30 seconds continuously for 15 min at

excitation or emission wavelengths of 395 or 495 nm, respectively. Stabilized fluorescence intensities measured during the last minute for each sample were averaged and presented as a bar graph. Each data bar represents the mean ±SD of two intensity measurements for n=3 independent wells during the last minute of each time course.

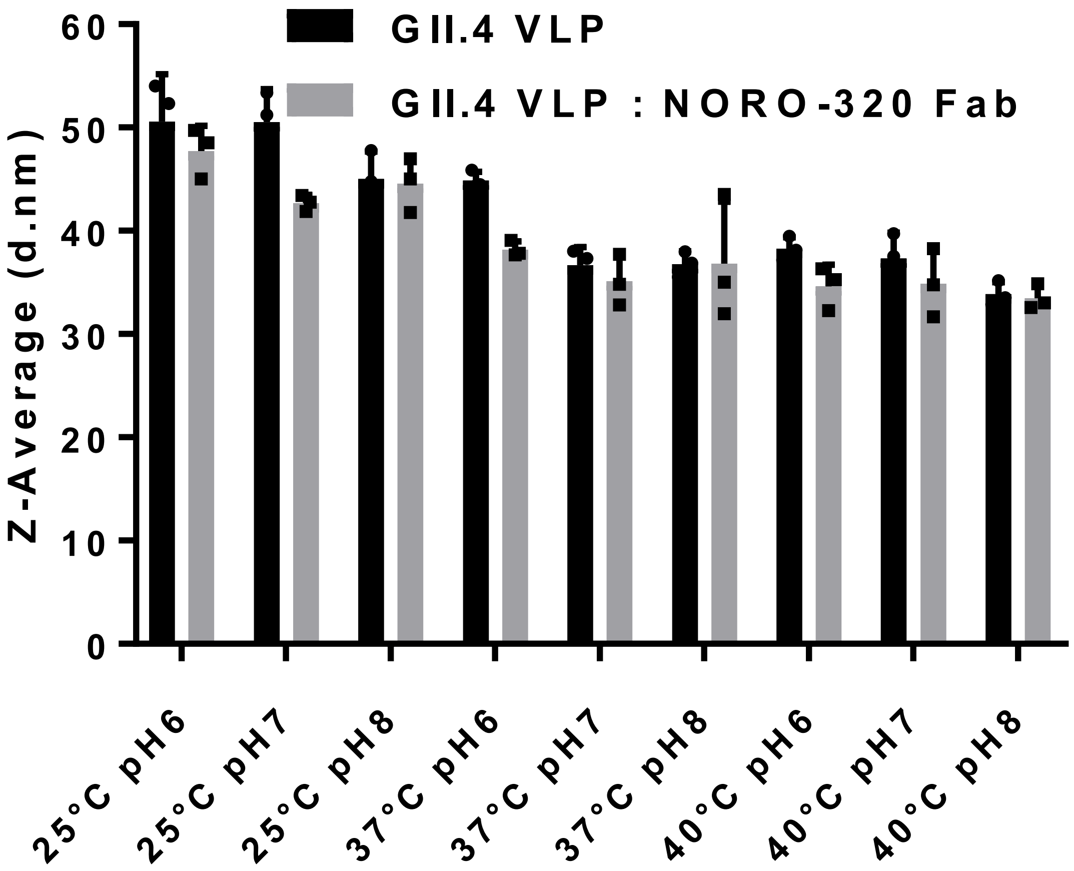

Supplemental Figure 4. Dynamic light scattering of mAb NORO-320 and GII.4 VLP with temperature and pH variation. Samples were diluted to a final concentration of 330 nM for each component in phosphate-buffered saline pH 6, pH 7, or pH 8 and incubated at the designated temperature for 30 minutes. Three × 12 measurement runs were performed with standard settings (refractive index 1.335, viscosity 0.9, temperature 25°C, 37°C, or 40°C). Each data bar represents the mean ±SD of n=3 independent experiments. The hydrodynamic diameters of treated or untreated GII.4 HuNoV VLPs were measured using dynamic light scattering (DLS) on a ZetaSizer Nano instrument (Malvern Instruments, UK). Z-Average hydrodynamic diameters were calculated for each sample condition using Zetasizer software.

Supplemental Tables

Supplemental Table 1. Antibody sequence analysis for 13 cross reactive human mAbs. Heavy and variable gene regions were sequenced. All of the mAbs had unique heavy and light chain complementary-determining region 3 (CDR3) sequences.

		Heavy Chain							Light Chain					
NORO mAb clone	Isotype	V gene	V gene % identity	J gene	J gene % identity	D gene	JUNCTION	HCDR length	V gene	V gene % identity	J gene	J gene % identity	AA JUNCTION	LCDR length
155.5	IgM	V4-34*01 F	100	J6*02 F	96.8	D 3-10*01 F	CARGLNTMVRGVLNYYYYYGMDVW	8.7.22	V1-6*01 F	100	J1*01 F	97.3	CLQDYNYPRTF	6.3.9
156.3	IgM	V4-34*01 F	100	J6*02 F	89.1	D3-16*01 F	CARGLMDVW	8.7.7	V1-8*01 F	100	J2*02 F	100	CQQYYSYPRTF	6.3.9
168.2	IgM	V3-30*01 F	100	J6*02 F	90.3	D3-10*01 F	CARSVIGYYYYGMDVW	8.8.14	V9-49*01	100	J2*01 F	89.5	CGADHGSGILF	7.8.9
170.5	IgM	V1-18*01 F	100	J1*01 F	90.4	D4-23*01 ORF	CARDQYGGNMYFQHW	8.8.13	V1-8*01 F	100	J5*01 F	100	CQQYYSYPITF	6.3.9
167.3	IgG	V5-51*01 F	100	J4*02 F	87.5	D2-15*01 F	CARSGRGHRGGSPDYW	8.8.14	V3-11*01 F	100	J3*01 F	97.4	CQQRSNWPFTF	6.3.9
178.6	IgG	V3-30*03 F	100	J4*02 F	85.2	D 6-19*01 F	CAKDWYLAMAGAAFDSW	8.8.15	V3-19*01 F	96.1	J2*01 F	81.6	CNSRDSSGKPSF	6.3.10
202A.2	IgG	V1-2*02 F	93.3	J3*01 F	92.0	D7-27*01 F	CARDLLRNWGDHDAFDVW	8.7.16	V1-47*01 F	97.5	J2*01 F	100	CSAWDDSLSGPV	1.3.11
279A	IgG	V3-30*03 F	91.7	J6*02 F	79.0	D2-8*01 F	CAKVEIHYYSNSLLGMDVW	8.8.17	V1-47*01 F	92.6	J3*02 F	94.6	CATLDINMTWVF	8.3.10
310A	IgG	V3-66*01 F	94.4	J4*02 F	93.8	D3-22*01 F	CTRDPSQYYDSRGHYYQTFTPSFDSW	8.7.24	V1-17*03 F	98.0	J4*01 F	100	CLQHDTYPLTF	6.3.9
323A	IgG	V3-30*03 F	95.8	J4*02 F	93.8	D1-14*01 ORF	CAKPVLSPFDYW	8.8.10	V2-14*01 F	97.2	J2*01 F	94.6	CCSYTSSSTEVF	9.3.10
232A.2	IgA	V4-34*02 F	83.5	J6*02 F	79.0	D 3-10*01 F	CARGRPHDYSPGSYSRPRRYYGLDVW	8.7.24	V3-20*01 F	91.8	J2*01 F	89.5	CQQYSSSPYTF	7.3.9
320	IgA	V1-69*01 F	95.5	J6*02 F	77.4	D3-10*01 F	CARDRVPSYSPSRRFSTKGAMWGKYGMDVW	8.8.28	V2-25*01 F	99.3	J1*01 F	100	CMQALQTPRTF	11.3.9
