# Supplementary material for: Broadly cross-reactive human antibodies that inhibit genogroup I and II noroviruses
Source: Nat Commun. 2021 Jul 14;12:4320. doi: 10.1038/s41467-021-24649-w (PMC8280134; doi:10.1038/s41467-021-24649-w)
Supplement: Supplementary file 3 — Reporting Summary [file 41467_2021_24649_MOESM3_ESM.pdf]

nature research

Corresponding author(s): James E. Crowe, Jr. and B. V. Venkataram Prasad

Last updated by author(s): May 27, 2021

Reporting Summary

Nature Research wishes to improve the reproducibility of the work that we publish. This form provides structure for consistency and transparency in reporting. For further information on Nature Research policies, see our Editorial Policies and the Editorial Policy Checklist.

Statistics

For all statistical analyses, confirm that the following items are present in the figure legend, table legend, main text, or Methods section.

n/a	Confirmed	
☐	☒	The exact sample size (*n*) for each experimental group/condition, given as a discrete number and unit of measurement
☐	☒	A statement on whether measurements were taken from distinct samples or whether the same sample was measured repeatedly
☐	☒	The statistical test(s) used AND whether they are one- or two-sided<br>*Only common tests should be described solely by name; describe more complex techniques in the Methods section.*
☒	☐	A description of all covariates tested
☐	☒	A description of any assumptions or corrections, such as tests of normality and adjustment for multiple comparisons
☒	☐	A full description of the statistical parameters including central tendency (e.g. means) or other basic estimates (e.g. regression coefficient) AND variation (e.g. standard deviation) or associated estimates of uncertainty (e.g. confidence intervals)
☒	☐	For null hypothesis testing, the test statistic (e.g. *F*, *t*, *r*) with confidence intervals, effect sizes, degrees of freedom and *P* value noted<br>*Give P values as exact values whenever suitable.*
☒	☐	For Bayesian analysis, information on the choice of priors and Markov chain Monte Carlo settings
☒	☐	For hierarchical and complex designs, identification of the appropriate level for tests and full reporting of outcomes
☒	☐	Estimates of effect sizes (e.g. Cohen's *d*, Pearson's *r*), indicating how they were calculated

Our web collection on statistics for biologists contains articles on many of the points above.

Software and code

Policy information about availability of computer code

Data collection	*Provide a description of all commercial, open source and custom code used to collect the data in this study, specifying the version used OR state that no software was used.*
Data analysis	GraphPad Prism v7.0

For manuscripts utilizing custom algorithms or software that are central to the research but not yet described in published literature, software must be made available to editors and reviewers. We strongly encourage code deposition in a community repository (e.g. GitHub). See the Nature Research guidelines for submitting code & software for further information.

Data

Policy information about availability of data

All manuscripts must include a data availability statement. This statement should provide the following information, where applicable:

- Accession codes, unique identifiers, or web links for publicly available datasets
- A list of figures that have associated raw data
- A description of any restrictions on data availability

Atomic coordinates and structure factors for the crystal structure of the NORO-320 Fab in complex with GII.4 P-domain has been deposited in the Protein Data Bank with the accession code 7JIE. The authors declare that all other data supporting the findings of this study are available within the paper and its supplementary information files.

Field-specific reporting

Please select the one below that is the best fit for your research. If you are not sure, read the appropriate sections before making your selection.

- [x] Life sciences
- [ ] Behavioural & social sciences
- [ ] Ecological, evolutionary & environmental sciences

For a reference copy of the document with all sections, see nature.com/documents/nr-reporting-summary-flat.pdf

Life sciences study design

All studies must disclose on these points even when the disclosure is negative.

Sample size	No samples size calculations were necessary for this study.
Data exclusions	No data was excluded.
Replication	For binding and blocking studies, technical replicates were included in each assay and experiments were repeated at least twice.
Randomization	This is not relevant to our study.
Blinding	Sample blinding was not relevant to this study.

Reporting for specific materials, systems and methods

We require information from authors about some types of materials, experimental systems and methods used in many studies. Here, indicate whether each material, system or method listed is relevant to your study. If you are not sure if a list item applies to your research, read the appropriate section before selecting a response.

Materials & experimental systems

n/a	Involved in the study	
	x	Antibodies
	x	Eukaryotic cell lines
x		Palaeontology and archaeology
x		Animals and other organisms
	x	Human research participants
x		Clinical data
x		Dual use research of concern

Methods

n/a	Involved in the study	
x		ChIP-seq
x		Flow cytometry
x		MRI-based neuroimaging

Antibodies

Antibodies used	Goat Anti-Human Kappa-HRP (Southern Biotech #2060-05); Goat Anti-Human Lambda-HRP (Southern Biotech #2070-05); Goat Anti-Mouse IgG, Human ads-HRP (Southern Biotech #1030-05)
Validation	Validation assurance was provided by the vendor.

Eukaryotic cell lines

Policy information about cell lines

Cell line source(s)	Gibco Sf9 cells; Thermo Fisher Scientific ExpiCHO cells
Authentication	None of the cell lines were authenticated
Mycoplasma contamination	Cell lines were tested for mycoplasma contamination on a monthly basis when in culture, and in every case found to be negative.
Commonly misidentified lines (See ICLAC register)	No commonly misidentified cell lines were used in this study

Human research participants

Policy information about studies involving human research participants

Population characteristics	The 6 adult individual subjects had a previous history of acute gastroenteritis but were otherwise healthy.
Recruitment	Participants were recruited based on a history of acute gastroenteritis.
Ethics oversight	Vanderbilt University Medical Center Institutional Review Board approved the protocol used in this study

Note that full information on the approval of the study protocol must also be provided in the manuscript.
